# Administration of Sodium Bicarbonate in Critically Ill Newborns: A Systematic Review and Meta-Analysis

**DOI:** 10.3390/jpm16010026

**Published:** 2026-01-05

**Authors:** Giovanni Boscarino, Susanna Esposito, Gianluca Terrin

**Affiliations:** 1 Pediatric Clinic, University Hospital, Department of Medicine and Surgery, University of Parma, 43126 Parma, Italy; giovanni.boscarino@unipr.it (G.B.); susannamariaroberta.esposito@unipr.it (S.E.); 2 Department of Maternal and Child Health, Policlinico Umberto I, Sapienza University of Rome, 00161 Rome, Italy

**Keywords:** mortality, neonatology, intraventricular hemorrhage, pulmonary hemorrhage, neurodevelopment, critical care, metabolic acidosis

## Abstract

**Background**: Metabolic acidosis is a frequent and serious complication in critically ill neonates, particularly preterm infants, and is associated with an increased risk of mortality, intraventricular hemorrhage, and long-term neurodevelopmental impairment. Despite limited evidence, sodium bicarbonate (SB) is widely administered in neonatal intensive care units (NICUs) to correct acidosis, largely extrapolated from adult and pediatric practice. However, concerns have been raised about its potential adverse effects, including paradoxical intracellular acidosis, impaired cerebral autoregulation, and increased risk of neurological injury. Given the uncertainty regarding both its efficacy and safety, we conducted a systematic review and meta-analysis to evaluate the role of SB administration in the neonatal population. **Methods**: MEDLINE, Scopus, and the Cochrane Library were searched using specific medical subject headings and terms. We included all study published up to July 2025 that involved newborns treated with SB. The primary outcome was positive response to treatment, while secondary outcomes included mortality, morbidity, and long-term impairment. **Results**: We analyzed 10 studies (9 randomized and 1 unrandomized study, including 660 neonates). Pooled results from the randomized controlled studies showed no efficacy of SB in newborns. Data from one unrandomized study showed an increased risk for mortality (OR 13.1 *p* = 0.02), clinical seizures (OR 2.8, *p* = 0.01), and a combined outcome of death or neurological damage (OR 3.1 *p* < 0.01) for neonates treated with SB. **Conclusions**: Current evidence is insufficient to support the routine administration of SB in NICUs. Neonatologists have the responsibility to administer only drugs of proven efficacy, personalizing therapy on the basis of a pathology’s etiology, in order to reduce risk and optimize benefits. In the absence of robust, statistically significant data, the indiscriminate use of SB should be discouraged in current clinical practice. PROSPERO registration number: CRD420251132502.

## 1. Introduction

Critically ill newborns, particularly preterm infants, are at high risk of hypoxia, impaired thermoregulation, and hypoperfusion. These conditions [1,2], associated with a limited bicarbonate reabsorption capacity of the kidneys, result in an accumulation of non-organic acids in the blood, with consequent metabolic acidosis (MA) [3]. MA can cause harm to preterm and full-term neonates at risk of neurodevelopmental delay [4,5]. It has been demonstrated that the occurrence of MA in preterm newborns during the first week of life significantly worsens neonatal outcomes. It has also been associated with bronchopulmonary dysplasia/death in extremely preterm infants [6].

In order to limit the severe cerebral and multi-organ damage associated with MA, since 1950, sodium bicarbonate (SB) has been used by neonatologists for resuscitation and treatment [4,5,7]. A Cochrane review published in 2005, including randomized controlled trials (RCTs) conducted between 1966 and January 2005, concluded that the available evidence for neonates was insufficient to support the routine use of SB in the treatment of MA [8]. From 2000 onwards, the use of SB has been removed from neonatal resuscitation guidelines [7,9,10]. Nevertheless, SB continues to be used in our NICUs to treat MA associated with other acute clinical syndromes, such as respiratory distress syndrome, hypoxia, and ongoing renal or gastrointestinal losses [7,11,12,13].

A survey published in 2021 (using data collected from 120 Italian NICUs between June 2017 and March 2018; response rate of 97.5%, 117/120 centers) showed that in 55% of the surveyed NICUs (64/117 units), it was common practice to correct MA using intravenous SB [14]. More recently, a multi-country survey found that 91.2% of neonatologists used SB to correct MA in the NICU, and 71.4% did not have written guidelines for its use [15]. Uncertainty persists regarding the safety of this practice. The administration of SB to reverse MA can lead to hemodynamic instability, arrhythmias, myocardial dysfunction, and disturbances in oxidative metabolism [16]. Studies on animal models have elucidated the mechanisms linking SB administration to myocardial depression [17]. Furthermore, SB can cause paradoxical respiratory acidosis, intracellular acidosis, electrolyte imbalances, and cerebrospinal fluid acidosis, all of which may contribute to neurological injury [16]. In neonates, intravenous infusions of SB have been reported to cause a transient paradoxical worsening of intracellular acidosis, loss of cerebral vascular autoregulation, reduced cerebral blood flow, and acute alterations in cerebrospinal fluid pH [18,19]. Furthermore, evidence from observational studies suggests that SB treatment during the first day of life is associated with an increased incidence of intraventricular hemorrhage (IVH) in very preterm infants [20].

In light of this, we conducted a systematic review and meta-analysis to evaluate the appropriateness of SB administration in newborns.

## 2. Materials and Methods

In accordance with the Preferred Reporting Items for Systematic Reviews and Meta-Analyses (PRISMA) guidelines [21] (Table S1), we conducted a systematic review and meta-analysis. We included all studies published up to July 2025 that involved newborns treated with SB in NICUs setting. The study protocol was registered in the PROSPERO database (CRD420251132502).

### 2.1. Research Methods, Study Selection, and Data Extraction

An electronic search was conducted in MEDLINE, Scopus, and the Cochrane Library using specific medical subject headings (MeSH) and terms (Table S2). Only studies published in English were considered eligible, including controlled and uncontrolled trials, such as observational studies and retrospective studies, involving newborns treated with SB in a NICU setting. Case reports and case series were excluded. One author (G.B.) assessed the eligibility of the studies based on predefined criteria and checked for duplicates. Duplicates were removed and the titles and/or abstracts of the remaining studies were independently screened by two reviewers (G.B. and G.T.) to identify those that met the inclusion criteria. Full texts of potentially eligible articles were then retrieved and independently assessed for eligibility by the same two reviewers, who were blinded to each other’s decisions. Any discrepancies were resolved through discussion with a third reviewer (SE) to achieve consensus. A manual search of the reference lists of all included articles, systematic reviews, and meta-analyses was also conducted.

For each selected study, data were extracted using a standardized form including authorship, year of publication, inclusion criteria, details of the intervention, and reported outcomes (efficacy on resolution of MA, mortality rate, intraventricular hemorrhage (IVH), pulmonary hemorrhage, neurodevelopmental (NDV) impairment, or other outcomes). Extracted data were verified for completeness, consistency, and accuracy. The corresponding authors of the selected studies were contacted when information was missing or unclear.

### 2.2. Outcome

The primary outcome was the efficacy and safety of the treatment. The definitions of efficacy varied between studies; thus, we used the one specified in each individual trial as dichotomous data. We considered safety to be the rate of mortality, IVH, or pulmonary hemorrhage.

The secondary outcome was the other safety outcomes evaluated in the included trials, such as NDV impairment, necrotizing enterocolitis (NEC), sepsis, and cardiological outcomes.

### 2.3. Risk of Bias

For controlled studies, we evaluated multiple domains of bias, including selection bias (adequacy of random sequence generation and allocation concealment), performance bias (blinding of study personnel regarding the intervention administered to each neonate), detection bias (blinding of outcome assessors), attrition bias (completeness of outcome data and the rationale and balance of missing data across study arms), reporting bias (consistency between prespecified outcomes and reported outcomes relevant to this review), and other potential biases (e.g., premature trial termination driven by interim analyses or methodological limitations inherent to the study design). Each study was assigned a risk of bias rating—high, low, or unclear—according to established criteria [22].

For uncontrolled studies, assessment of randomization and allocation concealment was not applicable. In these studies, the risk of selection bias was classified as low or high based on whether participant inclusion followed consecutive enrollment according to a predefined protocol and whether the number of excluded participants and reasons for exclusion were explicitly documented. When such information was insufficient or unavailable, the risk of selection bias was deemed unclear. Performance, detection, attrition, and reporting biases, as well as other sources of bias, were evaluated using the same criteria applied to controlled studies. Risk of bias assessments were performed independently by two reviewers (G.B. and G.T.) using a standardized data collection form. Discrepancies were resolved through discussion to achieve consensus. When key information required for risk of bias evaluation was incomplete or ambiguous in the published reports, corresponding authors were contacted for clarification.

### 2.4. Statistics

We used the Mantel–Haenszel method to calculate pooled odds ratios (ORs) and corresponding 95% confidence intervals (95%CIs) for dichotomous outcomes. Heterogeneity was assessed using Cochran’s Q test and quantified using the I^2^ statistic. A fixed-effects model was applied when heterogeneity was low (I^2^ ≤ 30%), while a random-effects model was used when I^2^ exceeded 30% [22]. For heterogeneity testing, a *p*-value of <0.1 was considered statistically significant; for comparison between the treatment groups, a *p*-value of <0.05 was considered statistically significant [22]. All analyses were conducted on an intention-to-treat basis using Review Manager (RevMan), version 5.4 (The Cochrane Collaboration, London, UK).

## 3. Results

### 3.1. Studies’ Selection and Characteristics

**Figure 1:**
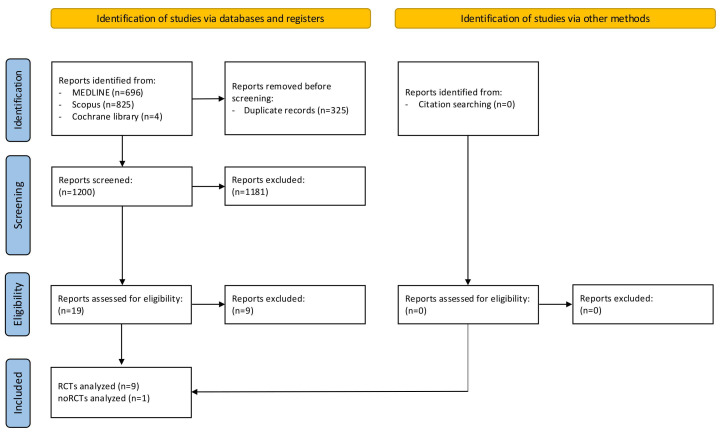
PRISMA flow-chart. Figure legend: randomized controlled trials (RCTs).

In the first review process, which involved searching databases and registers, we identified 1525 studies (Figure 1) that met our inclusion criteria. We removed 325 duplicate reports. After the screening process, 19 studies were considered eligible (Figure 1). The process of study identification conducted via other methods found no articles in the reference lists of eligible studies (Figure 1). Thus, in the final step of this meta-analysis, we excluded 9 studies, and we included 10 studies (9 RCTs and 1 retrospective study) [23,24,25,26,27,28,29,30,31,32].

The main characteristics of the included studies are reported in Table 1.

The outcomes of the selected studies are reported in Table 2 (for RCTs, treatment vs. control), Table 3 (for RCTs, different protocols of treatment), and Table 4 (for unrandomized controlled trials, treatment vs. control). As reported in Table 1, eight RCTs and one retrospective study compared a treated group (SB) with control groups [23,24,25,26,27,28,29,30,32], while one RCT compared two different protocols of administration of SB (slow vs. rapid infusion) [31]. No unrandomized controlled trials comparing two different treatment protocols were found.

### 3.2. Primary Outcome

#### 3.2.1. Evidence from Randomized Controlled Trials

**Figure 2:**
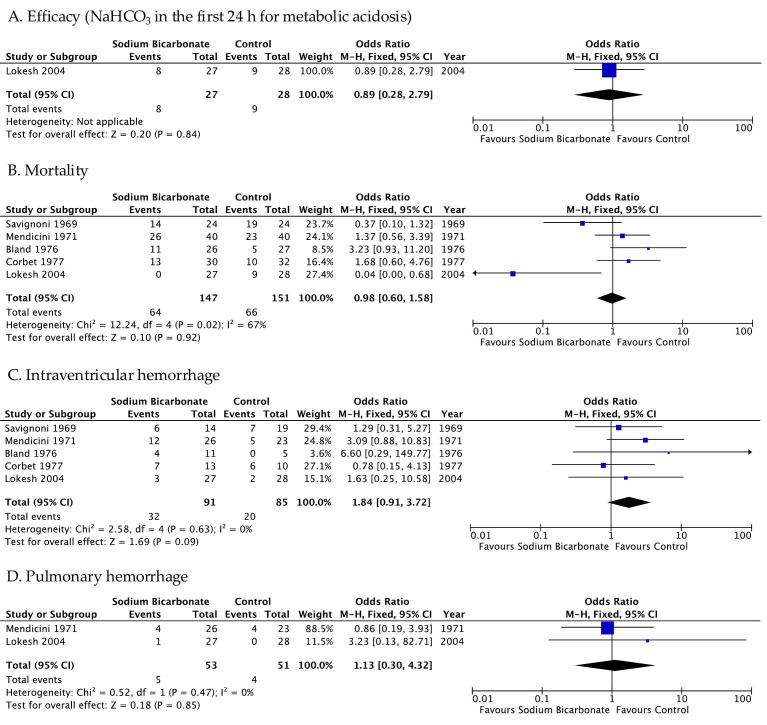
Outcomes of included randomized controlled trials [23,24,25,26,30].

Pooled results from the five RCTs that compared a treatment group vs. a control group showed no difference between the two groups in terms of efficacy, mortality, IVH, and pulmonary hemorrhage (Figure 2) [23,24,25,26,30]. It should be noted that only one study presented dichotomous data for the efficacy outcome (Figure 2A) [30] and that three RCTs did not show dichotomous outcomes for efficacy, mortality, IVH, or pulmonary hemorrhage [27,28,29]. Additionally, there is a marginally insignificant relationship between the rate of IVH and SB administration (OR 1.84 95% CI 0.91–3.72, *p* value 0.09—Figure 2B).

The only RCT comparing two different treatment protocols did not present dichotomous data on the efficacy outcome. The authors also did not evaluate the rates of mortality, IVH, and pulmonary hemorrhage. Therefore, we could not analyze the results (Table 3) [31].

We also intended to perform subgroup analyses including only infants born before 32 weeks’ gestation or with very low birth weight. However, this was not feasible because the study populations were too heterogeneous and inconsistently defined. Such an analysis would likely have included only one study, which would have had negligible statistical relevance.

#### 3.2.2. Evidence from Unrandomized Controlled Trials

**Figure 3:**
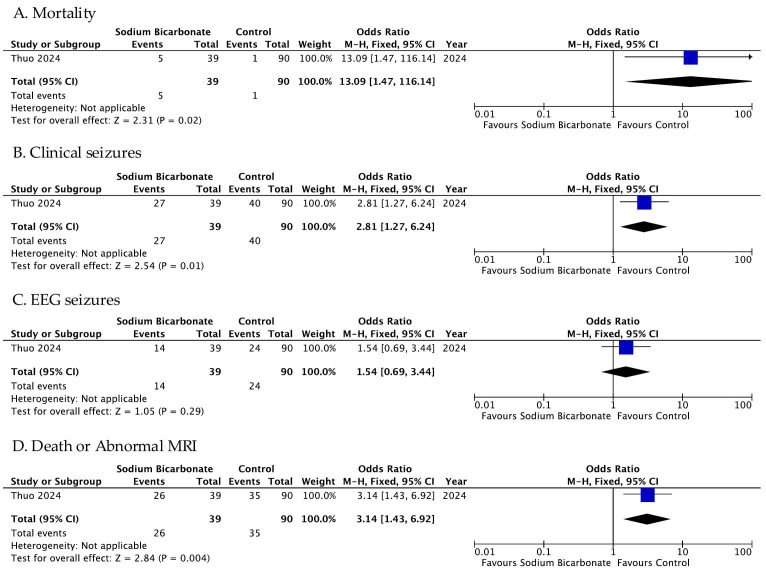
Outcome of included unrandomized trials. Figure legend: MRI (Magnetic Resonance Imaging) [32].

Analyzing the study of Thuo et al., we found a risk of mortality of 13.09 (95% CI 1.47–116.14, *p* value 0.02) for babies treated with SB compared with babies in the control group (Figure 3) [32]. The authors did not present dichotomous data on the efficacy outcome, and they did not evaluate the rates of IVH and pulmonary hemorrhage (Table 4).

### 3.3. Secondary Outcome

#### 3.3.1. Evidence from Randomized Controlled Trials

The cardiological outcome was evaluated only by Lokesh et al., who specifically described the rate of need for volume expansion (10 mL/kg of normal saline) for poor circulatory status, inotropic support, and persistent pulmonary hypertension [30]. However, we did not find a statistically significant relationship between the treatment and risk of a worse cardiological outcome (Figure S1).

Corbet et al. evaluated the rate of NEC and sepsis, but for these outcomes, too, there were no statistically significant differences between the treatment group and the control group (Figure S2) [26].

Neurological outcomes were evaluated by Mendicini et al. and Lokesh et al., but the authors evaluated different outcomes [24,30]. Mendicini et al. evaluated the rate of cloni, seizures, and increased tonus, while Lokesh et al. evaluated cerebral edema on ultrasonography, encephalopathy, and neurological abnormality at discharge. Our meta-analysis did not show a statistically significant relationship between these neurological outcomes and SB treatment (Figure S3).

#### 3.3.2. Evidence from Unrandomized Controlled Trials

As shown in Table 4 and Figure 3, Thuo et al. evaluated the rate of seizures separated into clinical and EEG seizures [32]. They found a risk of clinical seizures of 2.81 (95% CI 1.27–6.24, *p* value 0.01) for babies treated with SB; however, the relationship between the treatment and EEG seizures was not statistically significant (*p* value 0.29), as shown in Figure 3. Additionally, they evaluated a combined outcome of death and abnormal Magnetic Resonance Imaging (MRI) and found a risk of 3.14 (95% CI 1.43–6.92, *p* value 0.004) for babies in the treatment group (Figure 3).

### 3.4. Risk of Bias

**Figure 4:**
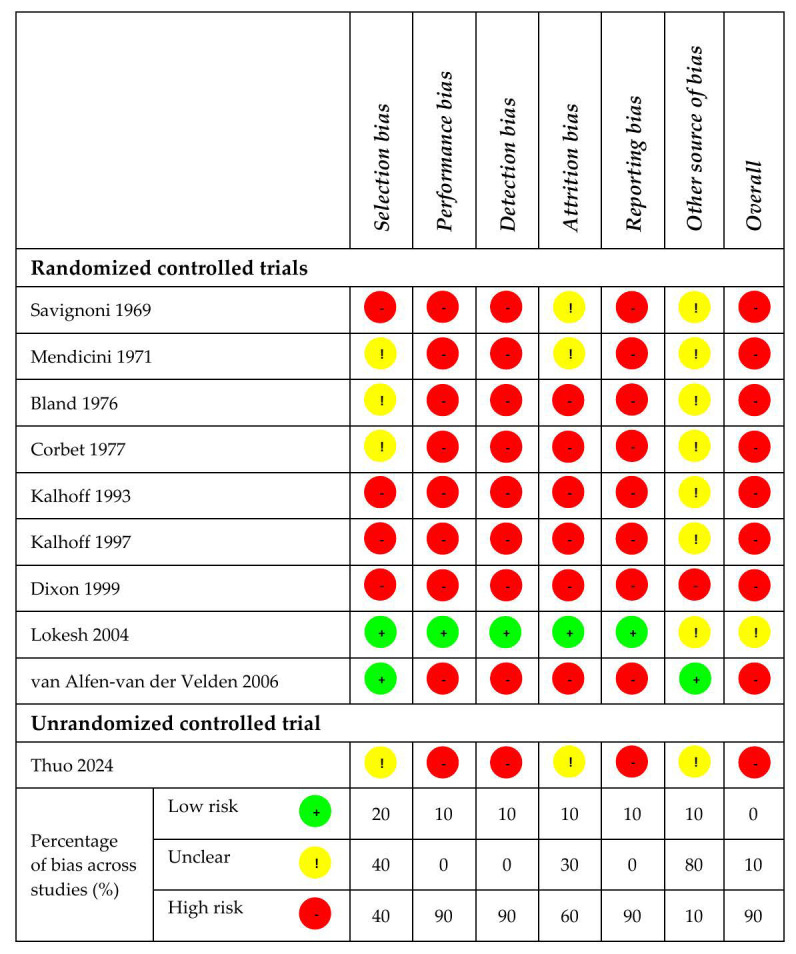
Risk of bias [23,24,25,26,27,28,29,30,31,32]. Yellow: unclear risk; red: high risk; green: low risk.

Risks of bias for the 10 included studies is reported in Figure 4. For selection bias, the risk was high in 40% of the studies, unclear in 40%, and low in 20%. The risk of performance bias, detection bias, and reporting bias was high in 90% of the studies and low in 10%. For attrition bias, the risk was evaluated high for 60%, unclear for 30%, and low for 10% of the studies. The other sources of bias were unclear for the majority of the studies (8/10, 80%), high for one study (10%), and low for one study (10%). Considering all the types of bias, the overall risk of bias was high for 90% and unclear for 10% of the studies.

## 4. Discussion

Evidence from RCTs does not demonstrate the efficacy of administering SB for treating MA in preterm neonates. When we analyzed a non-randomized trial in a meta-analysis, we found an increased risk of mortality, clinical seizures, and abnormal MRI findings or death among infants treated with SB. Our systematic review and meta-analysis showed that the current evidence is insufficient to support the routine administration of SB in NICUs.

MA can arise from various pathological conditions, such as renal failure, infection, hemodynamic instability, or other organic causes, leading to an inadequate compensatory response in critically ill patients—an effect that is further exacerbated in preterm neonates by systemic immaturity [33]. It is associated with both acute and chronic organ injury [2,34,35,36].

In non-neonatal populations, it has been shown that the administration of SB does not improve hemodynamic outcomes or oxygenation. An RCT conducted in 1999 in adult patients [37] showed that SB administration transiently increased pulmonary capillary pressure (from 15 to 17 mmHg and from 14 to 17 mmHg, *p* < 0.001) and cardiac output (18% and 16%, *p* < 0.01). However, no differences were observed in mean arterial pressure or other hemodynamic parameters.

The potential adverse cardiac effects of SB may be related to the exacerbation of intracellular acidosis due to CO_2_ generation during buffering, extracellular fluid hypertonicity when administered as a hypertonic solution, volume overload, excessive metabolic alkalosis, increased production of organic acids, and acceleration of Na^+^–H^+^ exchange, leading to deleterious intracellular Na^+^ and Ca^2+^ accumulation [38,39]. Studies that evaluate these outcomes in the neonatal population in order to better define the potential risks and benefits of SB administration are still lacking. Among the studies included in our meta-analysis, only Mendicini et al. and Lokesh et al. assessed hemodynamic outcomes. Mendicini et al. reported no differences in blood pressure changes between groups during the first 4 days following the intervention [24]. Lokesh et al. evaluated the use of inotropes, the need for volume expansion, and the persistence of pulmonary hypertension, again finding no significant differences between treated and untreated groups [30]. Nevertheless, two studies characterized by heterogeneous outcomes are insufficient to adequately assess the hemodynamic effects of SB in the neonatal population.

The RCTs included in our systematic review and meta-analysis did not show significant differences between groups for the evaluated outcomes. However, the non-RCT reported a higher risk of death, clinical seizures, and combined outcome of death and/or neurological damage in neonates treated with SB. Thuo et al. enrolled late preterm infants (≥35 weeks’ GA) with moderate-to-severe hypoxic–ischemic encephalopathy [32]. The authors suggested that neurological injury and death or abnormal MRI findings might partly result from CO_2_ production caused by SB administration [32]. This leads to increased intracellular CO_2_ and a consequent “paradoxical” intracellular acidosis [40]. They also noted that, while increasing SB administration alongside aggressive ventilation might prevent hypercapnia and the associated paradoxical acidosis [32,41], this hypothesis could not be evaluated. The study included a small cohort, lacked a predefined sample size calculation, and employed a retrospective design that did not allow for standardized assessments or treatment algorithms. Long-term follow-up data were also missing. Furthermore, the small sample size prevented the evaluation of significant associations after adjusting for sex and diagnosis of asphyxia.

The studies included in the meta-analysis used different protocols for SB administration. However, Van Alfen-van Der Valden et al. compared two protocols of SB administration [31]. This should be considered when interpreting the results of our meta-analysis.

Based on the results of our meta-analysis, which considered both RCTs and non-RCTs, the administration of SB therapy does not appear to be safe in the neonatal setting. A more appropriate approach for managing newborns with MA may be to focus on the underlying cause, although this has not yet been definitively demonstrated. Therefore, clinicians should prioritize identifying and correcting the underlying cause of acidosis, since SB therapy may increase mortality by failing to address the root cause and potentially exacerbating the clinical condition.

The quality of the studies included in this meta-analysis was affected by several risks of bias. Suboptimal blinding and randomization methods were employed in the RCTs. Furthermore, most of the studies were outdated, having been published more than 30–40 years ago. Some outcomes, such as IVH or pulmonary hemorrhage, were evaluated only in infants who died following autopsy, because diagnostic tools such as cranial ultrasound were unavailable at that time. Specifically, we found a marginally insignificant relation between the occurrence of IVH and SB administration. Due to advances in clinical support techniques for these neonates, particularly the use of cranial ultrasound, it cannot be excluded that some IVHs may have gone undetected, which could have substantially altered the results. Therefore, this outcome, as well as other data assessed post-mortem, is not evaluable. In addition, most of the included studies were of poor methodological quality and enrolled a small number of patients, without a prespecified sample size calculation.

Our results should also be interpreted in light of some methodological limitations of the meta-analysis. During the review process, we attempted to identify studies using alternative methods, such as screening the reference lists of published reviews and articles, to minimize the risk of missing relevant data. However, we restricted inclusion to articles published in English, meaning that some data may have been overlooked. Furthermore, despite carefully analyzing the collected data, some outcomes—primarily those related to efficacy—could not be reliably assessed, as dichotomous data were either not reported or not derivable, leading to uncertainty about the true effectiveness of the intervention.

## 5. Conclusions

Based on our findings, there is currently no compelling evidence supporting the efficacy or safety of SB in neonatal care. Early therapeutic interventions in the first days of life can have important effects on both mortality and long-term outcomes, which remain central priorities in contemporary neonatal medicine. In this context, medical practice, especially in populations at exceptionally high risk of long-term sequelae, is increasingly oriented toward personalized treatment strategies, aiming to tailor interventions to the underlying etiology and the individual clinical profile. We believe that neonatologists have the responsibility to administer only drugs of proven efficacy, personalizing therapy on the basis of the etiology of the pathology, in order to reduce risk and optimize benefits.

In the absence of robust and statistically significant data, the indiscriminate use of SB should be discouraged, particularly in preterm infants, but also in critically ill term neonates.

## Figures and Tables

**Table 1:** Main characteristics of the included studies.

Study ID, Country	Inclusion Criteria	Intervention *n*	Control *n*
RCT			
Savignoni 1969, Italy [23]	- <24 h of life; - BW 1250–2500 g; - Silverman score > 2; - Reticulogranular pattern on chest film	NaHCO_3_ (mEq given = BE in mEq/L × BW in Kg × 0.5 in 2–6 h) + GS 10% (70 mL/kg/d) *n* = 24	GS 10% (25 mL/Kg/d) *n* = 24
Mendicini 1971, Italy [24]	- BW 750–1250 g;	NaHCO_3_ (mEq given = BE, mEq/L × BW in kg, × 0.5, given in 2–6 h + GS 10% (70 mL/kg/d) for at least 4 days *n* = 40	GS 10% (25 mL/kg/day) from 2nd to 5th day *n* = 40
Bland 1976, USA [25]	Hypoproteinemia (cord serum total protein level of 4.6 gm/100 mL or less) and GA < 37 wks	I1: 3 mL/kg of NaHCO_3_ and 5 mL/kg of water *n* = 13 I2: 1.5 mL/kg of NaHCO_3_, 4 mL/kg of salt-poor albumin, and 2.5 mL/kg of water *n* = 13 *n* total 26	C1: 8 mL/kg of glucose in water *n* = 13 C2: 8 mL/kg of salt-poor albumin *n* = 14 *n* total 27
Corbet 1977, Texas [26]	- BW < 1500 g; - GA ≤ 32; - Apgar score ≤ 3 at 1′; - Clinical diagnosis of HMD or BW < 2000 g and requiring assisted ventilation	NaHCO_3_ as following: pH 7.25–7.30, 5 mEq/dL; pH 7.15–7.25, 10 mEq/dL; pH < 7.15–15 mEq/dL + GS 10% (65 mL/kg/d) *n* = 30	GS 10% (65 mL/kg/d) *n* = 32 §
Kalhoff 1993, Germany [27]	Premature babies with BW ≤ 1.5 kg or SGA with BW ≥ 1.5 kg.	Oral NaHCO_3_ 2 mmol/kg/d for 7 days *n* = 77	No NaHCO_3_ *n* = 93
Kalhoff 1997, Germany [28]	Premature infants and SGA from 1.0 to 1.9 kg	Oral NaHCO_3_ 2 mmol/kg/d for 7 days *n* = 27	Oral NaCL 2 mmol/kg/d for 7 days *n* = 26
Dixon 1999, United Kingdom [29]	pH < 7.25 and BE worse than −6.	4.2% NaHCO_3_ at a dose in mmol of one-sixth × weight (kg) × BE infused over 30 min. *n* = 16	10 mL/kg 4.5% human albumin *n* = 20
Lokesh 2004, India [30]	Asphyxiated neonates continuing to need positive pressure ventilation at 5 min of life	NaHCO_3_ 4 mL/kg (1.8 meq/kg) over 3–5 min *n* = 27	4 mL/kg of GS 5% *n* = 28
van Alfen-van der Velden 2006, The Netherlands [31]	GA between 26 and 34 wks with metabolic acidosis	NaHCO_3_ administered within approximately 15 s (Rapid group) *n* = 15	NaHCO_3_ over a 30 min period (Slow group) *n* = 14
Retrospective cohort study			
Thuo 2024, USA [32]	≥35 wks of GA, with moderate or severe HIE	NaHCO_3_ *n* = 39	No NaHCO_3_ *n* = 90

Table legend: RCT (randomized controlled trial); GS (glucose solution); I (intervention group); C (control group); BW (birth weight); BE (base excess); GA (gestational age); HMD (hyaline membrane disease); SGA (small for gestational age); HIE (hypoxic–ischemic encephalopathy); § 6 infants received NaHCO_3_ in delivery room.

**Table 2:** Main outcomes of included RCTs that compared sodium bicarbonate vs. control group.

Study ID	Efficacy (Resolution of MA)	Mortality	IVH	PH	NDV Impairment	Other
Savignoni 1969 [23]	Not available dichotomous data	I: 14 vs. C: 19 *p* value = 0.076	I: 6 vs. C: 7 (post-mortem) *p* value ND	/	/	/
Mendicini 1971 [24]	/	I: 26 vs. C: 23 *p* value > 0.05	I: 12 vs. C: 5 (post-mortem) *p* value = 0.07	I: 4 vs. C: 4 (post-mortem) *p* value > 0.05	Cloni I: 2 vs. C: 5 Increased tonus I: 0 vs. C: 4 Seizures I: 0 vs. C: 0 *p* value > 0.05	No difference in BP between the two group, in the first 4 days of life
Bland 1976 [25]	Not available dichotomous data	I1: 6 I2: 5 C1: 1 C2: 4 I total: 11 C total: 5 *p* value ND	I1: 3 I2: 1 C1: 0 C2: 0 I total: 4 C total: 0 (post-mortem) *p* value ND	/	/	/
Corbet 1977 [26]	Not available dichotomous data	I: 13 vs. C: 10 *p* value > 0.05	I: 7 vs. C: 6 (post-mortem) *p* value > 0.5	/	/	NEC I: 1 vs. C: 0 (post-mortem) *p* value ND Sepsis I: 3 vs. C: 1 (post-mortem) *p* value ND
Kalhoff 1993 [27]	Not available dichotomous data	ND	ND	/	/	/
Kalhoff 1997 [28]	Not available dichotomous data	ND	ND	/	/	/
Dixon 1999 [29]	Not available dichotomous data	ND	ND	/	/	/
Lokesh 2004 [30]	Need of NaHCO_3_ in the first 24 h I: 8 vs. C: 9 *p* value > 0.05	I: 0 vs. C: 9 *p* value 0.84	I: 3 vs. C: 2 *p* value > 0.05	I: 1 vs. C: 0 *p* value > 0.05	Neurological abnormality at discharge I: 5 vs. C: 6 (Percentage out of survivors) *p* value 0.10 Encephalopathy I: 20 vs. C: 18 *p* value > 0.05 Cerebral edema on ultrasonography I: 14 vs. C: 9 *p* value > 0.05	Need for volume expansion I: 10 vs. C: 8 *p* value > 0.05 Inotropic support I: 12 vs. C: 8 *p* value > 0.05 PPHN I: 1 vs. C: 0 *p* value > 0.05

Table legend: RCT (randomized controlled trial); MA (metabolic acidosis); IVH (intraventricular hemorrhage); PH (pulmonary hemorrhage); NDV (neurodevelopmental); I (intervention group); C (control group); BP (blood pressure); NEC (necrotizing enterocolitis); PPHN (persistent pulmonary hypertension); ND (not declared).

**Table 3:** Main outcomes of included RCT that compared two different protocols of sodium bicarbonate administration.

Study ID	Efficacy (Resolution of MA)	Mortality	IVH	PH	NDV Impairment	Other
van Alfen-van der Velden 2006 [31]	Not available dichotomous data	/	/	/	/	Infusion of NaHCO_3_ resulted in a statistically significant increase in CBV over time in both groups (*p* < 0.001 rapid group; *p* < 0.01 slow group).

Table legend: RCT (randomized controlled trial); MA (metabolic acidosis); IVH (intraventricular hemorrhage); PH (pulmonary hemorrhage); NDV (neurodevelopmental); CBV (cerebral blood volume).

**Table 4:** Main outcome of included unrandomized controlled trial that compared sodium bicarbonate vs. control group.

Study ID	Efficacy (Resolution of MA)	Mortality	IVH	PH	NDV Impairment	Other
Thuo 2024 [32]	Not available dichotomous data	I: 5 vs. C: 1 *p* value 0.010	/	/	Clinical Seizures I: 27 vs. C: 40 *p* value 0.013	/
					EEG seizures I: 14 vs. C: 24 *p* value 0.408	
					Death or Abnormal MRI: I: 26 vs. C: 35 *p* value 0.004	

Table legend. MA (metabolic acidosis); IVH (intraventricular hemorrhage); PH (pulmonary hemorrhage); NDV (neurodevelopmental); I (intervention group); C (control group); EEG (electroencephalogram); MRI (Magnetic Resonance Imaging).

## Data Availability

The original contributions presented in this study are included in the article and Supplementary Materials. Further inquiries can be directed to the corresponding author.

## Supplementary Materials

The following supporting information can be downloaded at https://www.mdpi.com/article/10.3390/jpm16010026/s1, Table S1: PRISMA checklist; Table S2: Medical subject headings and terms used for electronic search; Figure S1: Cardiological outcomes derived from randomized controlled trials [30]; Figure S2: Other outcomes derived from randomized controlled trials [26]; Figure S3: Neurological outcomes derived from randomized controlled trials [24,30].

## Abbreviations

The following abbreviations are used in this manuscript:

BE	Base excess
BP	Blood pressure
BW	Birth weight
CBV	Cerebral blood volume
EEG	Electroencephalogram
GA	Gestational age
HIE	Hypoxic–ischemic encephalopathy
HMD	Hyaline membrane disease
IVH	Intraventricular hemorrhage
MeSH	Medical subject headings
MA	Metabolic acidosis
MRI	Magnetic resonance imaging
NDV	Neurodevelopmental
ND	Not declared
NEC	Necrotizing enterocolitis
NICUs	Neonatal intensive care units
RCTs	Randomized controlled trials
SB	Sodium bicarbonate
SGA	Small for gestational age
PRISMA	Preferred Reporting Items for Systematic Reviews and Meta-Analyses
95% CI	95% confidence interval
