# Supplementary material for: Administration of Sodium Bicarbonate in Critically Ill Newborns: A Systematic Review and Meta-Analysis
Source: J Pers Med. 2026 Jan 5;16(1):26. doi: 10.3390/jpm16010026 (PMC12842761; doi:10.3390/jpm16010026)

Figure S1. Cardiological outcome derived from randomized controlled trials.

A. Need of volume expansion (10 ml/kg of normal saline) for poor circulatory status

Study or Subgroup	Sodium Bicarbonate Events	Sodium Bicarbonate Total	Control Events	Control Total	Weight	Odds Ratio M-H, Fixed, 95% CI	Year
Lokesh 2004	10	27	8	28	100.0%	1.47 [0.47, 4.56]	2004
**Total (95% CI)**		**27**		**28**	**100.0%**	**1.47 [0.47, 4.56]**	
Total events	10		8				

Heterogeneity: Not applicable
Test for overall effect: Z = 0.67 (P = 0.50)

Odds Ratio M-H, Fixed, 95% CI

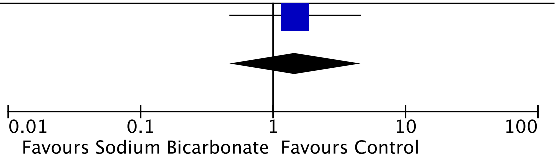

B. Inotropic support

Study or Subgroup	Sodium Bicarbonate Events	Sodium Bicarbonate Total	Control Events	Control Total	Weight	Odds Ratio M-H, Fixed, 95% CI	Year
Lokesh 2004	12	27	8	28	100.0%	2.00 [0.65, 6.11]	2004
**Total (95% CI)**		**27**		**28**	**100.0%**	**2.00 [0.65, 6.11]**	
Total events	12		8				

Heterogeneity: Not applicable
Test for overall effect: Z = 1.22 (P = 0.22)

Odds Ratio M-H, Fixed, 95% CI

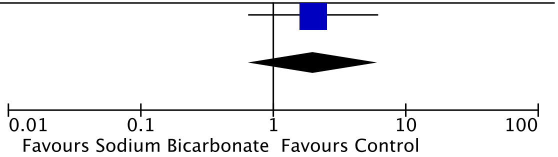

C. Persistent pulmonary hypertension

Study or Subgroup	Sodium Bicarbonate Events	Sodium Bicarbonate Total	Control Events	Control Total	Weight	Odds Ratio M-H, Fixed, 95% CI	Year
Lokesh 2004	1	27	0	28	100.0%	3.23 [0.13, 82.71]	2004
**Total (95% CI)**		**27**		**28**	**100.0%**	**3.23 [0.13, 82.71]**	
Total events	1		0				

Heterogeneity: Not applicable
Test for overall effect: Z = 0.71 (P = 0.48)

Odds Ratio M-H, Fixed, 95% CI

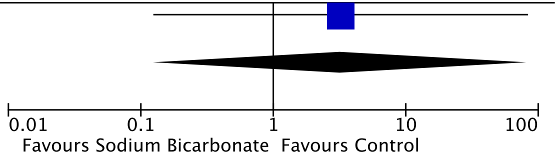

Supplement: Supplementary file 1 [file jpm-16-00026-s001.zip › Figure S1.pdf]
