# Supplementary material for: Administration of Sodium Bicarbonate in Critically Ill Newborns: A Systematic Review and Meta-Analysis
Source: J Pers Med. 2026 Jan 5;16(1):26. doi: 10.3390/jpm16010026 (PMC12842761; doi:10.3390/jpm16010026)

Figure S2. Other outcome derived from randomized controlled trials.

A. Necrotizing enterocolitis

	Sodium Bicarbonate		Control			Odds Ratio		Odds Ratio
Study or Subgroup	Events	Total	Events	Total	Weight	M-H, Fixed, 95% CI	Year	M-H, Fixed, 95% CI
Corbet 1977	1	13	0	10	100.0%	2.52 [0.09, 68.60]	1977	
**Total (95% CI)**		**13**		**10**	**100.0%**	**2.52 [0.09, 68.60]**		
Total events	1		0					
Heterogeneity: Not applicable								
Test for overall effect: Z = 0.55 (P = 0.58)								

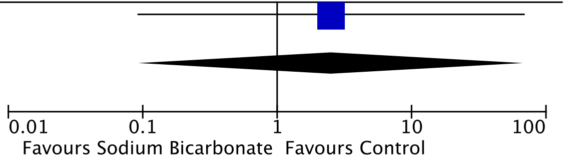

B. Sepsis

	Sodium Bicarbonate		Control			Odds Ratio		Odds Ratio
Study or Subgroup	Events	Total	Events	Total	Weight	M-H, Fixed, 95% CI	Year	M-H, Fixed, 95% CI
Corbet 1977	3	13	1	10	100.0%	2.70 [0.24, 30.85]	1977	
**Total (95% CI)**		**13**		**10**	**100.0%**	**2.70 [0.24, 30.85]**		
Total events	3		1					
Heterogeneity: Not applicable								
Test for overall effect: Z = 0.80 (P = 0.42)								

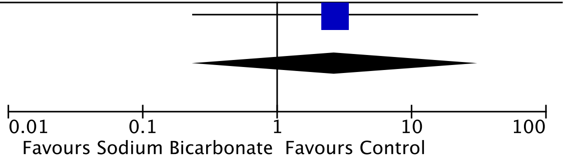

Supplement: Supplementary file 1 [file jpm-16-00026-s001.zip › Figure S2.pdf]
