# Supplementary material for: Administration of Sodium Bicarbonate in Critically Ill Newborns: A Systematic Review and Meta-Analysis
Source: J Pers Med. 2026 Jan 5;16(1):26. doi: 10.3390/jpm16010026 (PMC12842761; doi:10.3390/jpm16010026)

Figure S3. Neurological outcome derived from randomized controlled trials.

A. Cloni

Study or Subgroup	Sodium Bicarbonate Events	Sodium Bicarbonate Total	Control Events	Control Total	Weight	Odds Ratio M-H, Fixed, 95% CI	Year
Mendicini 1971	2	23	5	26	100.0%	0.40 [0.07, 2.30]	1971
**Total (95% CI)**		**23**		**26**	**100.0%**	**0.40 [0.07, 2.30]**	
Total events	2		5				
Heterogeneity: Not applicable							
Test for overall effect: Z = 1.03 (P = 0.30)							

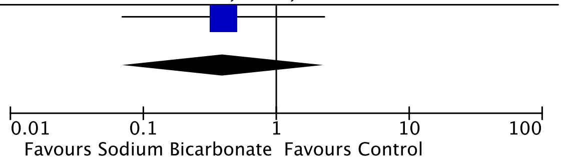

B. Seizures

Study or Subgroup	Sodium Bicarbonate Events	Sodium Bicarbonate Total	Control Events	Control Total	Weight	Odds Ratio M-H, Fixed, 95% CI	Year
Mendicini 1971	0	23	0	26		Not estimable	1971
**Total (95% CI)**		**23**		**26**		**Not estimable**	
Total events	0		0				
Heterogeneity: Not applicable							
Test for overall effect: Not applicable							

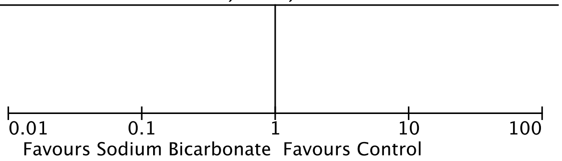

C. Cerebral oedema on ultrasonography

Study or Subgroup	Sodium Bicarbonate Events	Sodium Bicarbonate Total	Control Events	Control Total	Weight	Odds Ratio M-H, Fixed, 95% CI	Year
Lokesh 2004	14	27	9	28	100.0%	2.27 [0.76, 6.80]	2004
**Total (95% CI)**		**27**		**28**	**100.0%**	**2.27 [0.76, 6.80]**	
Total events	14		9				
Heterogeneity: Not applicable							
Test for overall effect: Z = 1.47 (P = 0.14)							

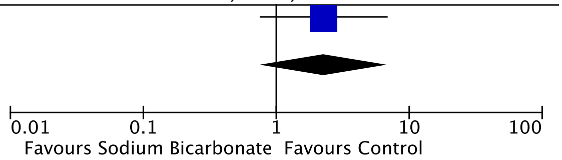

D. Encephalopathy

Study or Subgroup	Sodium Bicarbonate Events	Sodium Bicarbonate Total	Control Events	Control Total	Weight	Odds Ratio M-H, Fixed, 95% CI	Year
Lokesh 2004	20	27	18	28	100.0%	1.59 [0.50, 5.05]	2004
**Total (95% CI)**		**27**		**28**	**100.0%**	**1.59 [0.50, 5.05]**	
Total events	20		18				
Heterogeneity: Not applicable							
Test for overall effect: Z = 0.78 (P = 0.43)							

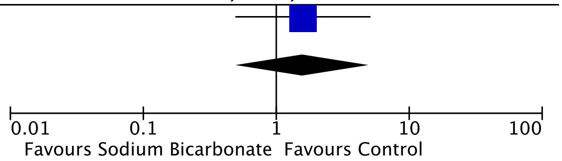

E. Increased tonus

Study or Subgroup	Sodium Bicarbonate Events	Sodium Bicarbonate Total	Control Events	Control Total	Weight	Odds Ratio M-H, Fixed, 95% CI	Year
Mendicini 1971	0	23	4	26	100.0%	0.11 [0.01, 2.09]	1971
**Total (95% CI)**		**23**		**26**	**100.0%**	**0.11 [0.01, 2.09]**	
Total events	0		4				
Heterogeneity: Not applicable							
Test for overall effect: Z = 1.47 (P = 0.14)							

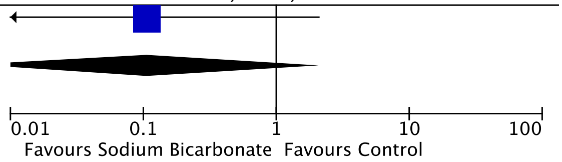

F. Neurological abnormality at discharge

Study or Subgroup	Sodium Bicarbonate Events	Sodium Bicarbonate Total	Control Events	Control Total	Weight	Odds Ratio M-H, Fixed, 95% CI	Year
Lokesh 2004	5	27	6	28	100.0%	0.83 [0.22, 3.14]	2004
**Total (95% CI)**		**27**		**28**	**100.0%**	**0.83 [0.22, 3.14]**	
Total events	5		6				
Heterogeneity: Not applicable							
Test for overall effect: Z = 0.27 (P = 0.79)							

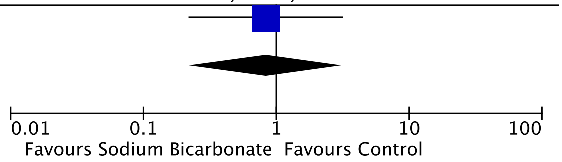

Supplement: Supplementary file 1 [file jpm-16-00026-s001.zip › Figure S3.pdf]
