# Supplementary material for: Administration of Sodium Bicarbonate in Critically Ill Newborns: A Systematic Review and Meta-Analysis
Source: J Pers Med. 2026 Jan 5;16(1):26. doi: 10.3390/jpm16010026 (PMC12842761; doi:10.3390/jpm16010026)
Supplement: Supplementary file 1 [file jpm-16-00026-s001.zip › Table S2.pdf]

Table S2. Medical subject headings and terms used for electronic search.

(((sodium bicarbonate) OR (NaHCO3)) AND (treatment) AND ((newborn) OR (neonate) OR (preterm) OR (neonatology) OR (infant)))
